# Taurine Rescues Cisplatin-Induced Muscle Atrophy In Vitro: A Morphological Study

**DOI:** 10.1155/2014/840951

**Published:** 2014-05-13

**Authors:** Alessandra Stacchiotti, Francesca Rovetta, Matteo Ferroni, Giovanni Corsetti, Antonio Lavazza, Giorgio Sberveglieri, Maria Francesca Aleo

**Affiliations:** ^1^Division of Anatomy and Physiopathology, Department of Clinical and Experimental Sciences, Brescia University, Viale Europa 11, 25123 Brescia, Italy; ^2^Department of Molecular and Translational Medicine, Brescia University, Viale Europa 11, 25123 Brescia, Italy; ^3^Department of Information Engineering, CNR-IDASC Sensor Laboratory, Brescia University, Via Valotti 9, 25123 Brescia, Italy; ^4^Istituto Zooprofilattico Sperimentale della Lombardia e Dell'Emilia-Romagna, Via A. Bianchi 7/9, 25124 Brescia, Italy

## Abstract

Cisplatin (CisPt) is a widely used chemotherapeutic drug whose side effects include muscle weakness and cachexia. Here we analysed CisPt-induced atrophy in C2C12 myotubes by a multidisciplinary morphological approach, focusing on the onset and progression of autophagy, a protective cellular process that, when excessively activated, may trigger protein hypercatabolism and atrophy in skeletal muscle. To visualize autophagy we used confocal and transmission electron microscopy at different times of treatment and doses of CisPt. Moreover we evaluated the effects of taurine, a cytoprotective beta-amino acid able to counteract oxidative stress, apoptosis, and endoplasmic reticulum stress in different tissues and organs. Our microscopic results indicate that autophagy occurs very early in 50 **μ**M CisPt challenged myotubes (4 h–8 h) before overt atrophy but it persists even at 24 h, when several autophagic vesicles, damaged mitochondria, and sarcoplasmic blebbings engulf the sarcoplasm. Differently, 25 mM taurine pretreatment rescues the majority of myotubes size upon 50 **μ**M CisPt at 24 h. Taurine appears to counteract atrophy by restoring regular microtubular apparatus and mitochondria and reducing the overload and the localization of autophagolysosomes. Such a promising taurine action in preventing atrophy needs further molecular and biochemical studies to best define its impact on muscle homeostasis and the maintenance of an adequate skeletal mass in vivo.

## 1. Introduction

Skeletal muscles are a plastic tissue that continuously renovates protein aggregates, exhausted organelles like mitochondria, and sarcoplasmic reticulum, to maintain an efficient metabolism [1]. Aging, starvation or anorexia, several toxic or inflammatory disorders, cancer, disuse, or immobilization all concur to activate excessive catabolism in muscle that exerts progressive atrophy [2, 3].

We previously reported that cisplatin (CisPt), a well-known chemotherapic drug for solid organs cancer and leukemia, was able to induce atrophy in vitro in murine C2C12 myotubes. In particular we demonstrated that CisPt, through Akt pathway impairment, behaved as a potent trigger to cause atrophy via ubiquitin-proteasome and autophagy-lysosome systems [4–6]. In skeletal muscle, macroautophagy, called hereafter autophagy, is considered a compensatory mechanism to eliminate abnormal proteins and organelles in order to maintain mass and prolong longevity [7, 8]. Morphologically autophagy is characterized by the generation of double membrane vesicles (phagophores) which encompass portion of cytoplasm, organelles, glycogen, and protein aggregates becoming autophagosomes and then degraded by lysosomal proteases upon mature autophagolysosomes [9, 10]. Taurine (2-aminoethanesulfonic acid) (Tau) is a free beta-amino acid distributed in mammals and human body, mainly in the brain, heart, kidney, and reproductive organs, with many physiological activities [11, 12]. In skeletal muscles Tau stabilizes phospholipids in sarcolemma, regulates Ca^2+^ ions flux, and limits exercise-induced weakness [13, 14]. Moreover, Tau supplementation modulates autophagy and reduces both endoplasmic reticulum (ER) stress and apoptosis induced by methamphetamine, glutamate, or CisPt, respectively, in neuronal or renal cell lines [15–17]. However, Tau impacts atrophic C2C12 myotubes treated with CisPt and a detailed morphological analysis of autophagy has been never analysed yet. Indeed to visualize autophagic structures in situ in skeletal muscles is a technically hard problem due to myofibers morphological peculiarity (i.e., to treat syncytia) and the small size of autophagic vesicles in muscles.

Due to this background the major aims of the current study were (1) to deeply describe by confocal, SEM, and TEM analysis the onset and progression of autophagy and atrophy in C2C12 myotubes exposed to CisPt and (2) to visualize the effects of Tau preconditioning on myotube size and autophagy. Our morphological results suggest that in cultured C2C12 myotubes taurine pretreatment rescues CisPt-induced myotubes atrophy and regulates the activity of the autophagy-lysosome system by maintaining proper perinuclear autophagic vesicles and mitochondria size and density.

## 2. Materials and Methods

### 2.1. Reagents

All chemicals, antibodies, and reagents were purchased from Sigma-Aldrich (Milan, Italy), if not otherwise indicated. Cell culture ware was purchased from Becton-Dickinson Falcon (Sacco srl, Milan, Italy).

### 2.2. Cell Culture

Mouse C2C12 myoblasts were grown in Dulbecco's modified Eagle's medium (DMEM) supplemented with 10% foetal bovine serum and 100 *μ*g/mL penicillin-streptomycin antibiotic in a humidified 5% CO_2_ atmosphere at 37°C. To induce differentiation 80% confluent cells were cultured in differentiating medium (DM) containing 2% horse serum for 5 days [18].

### 2.3. Drug Treatments

CisPt was dissolved in 1.5% DMSO to obtain 5 mM stock solution and administered to cells for 4 h up to 24 h at 10 or 50 *μ*M as previously reported [4]. Control experiments were conducted in parallel with equivalent amount of DMSO. Myotube starvation was obtained by culturing cells in 10 mM Hank's/Hepes buffer (pH 7.4) devoid of glucose and amino acids. Taurine preconditioning was performed by dissolving the powder at 25 mM in culture medium for 1 h before adding CisPt for above times. This pretreatment has been reported to be effective against toxicity induced by CisPt and glutamate in NRK52E renal cells and PC12 neurons [16, 19]. To inhibit autophagy, a specific inhibitor of early phases, 3-methyladenine (3-MA) 5 mM was added to DMSO or CisPt-treated cultures for up to 24 h [20].

### 2.4. Necrosis and Apoptosis Detection

Necrosis was assessed by the incorporation of propidium iodide (PI). Five-day-old differentiated myotubes were incubated for different time-points with 10 *μ*M and 50 *μ*M CisPt and then cotreated with 1 *μ*g/mL PI and Hoechst (Molecular Probes) to stain nuclei. After 30 min of incubation at 37°C, cells were washed in PBS buffer, fixed for 5 min in ice-cold 70% ethanol, and observed by fluorescence microscopy. Percentage of necrotic cells was expressed as the ratio between the number of cells with disrupted plasma membrane (i.e., PI-positive nuclei) and the total number of Hoechst-positive nuclei. Apoptosis was determined based on the increased sensitivity of apoptotic nuclei to DNA thermal denaturation. Briefly, 5-day-old myotubes cultured on coverglasses were treated for different time points with above concentrations of CisPt, fixed for 20 min in 4% paraformaldehyde, and then incubated for 20 min in 0.2% Triton X-100. Then, slides were exposed to 50% formamide (v/v distilled water), heated for 20 min at 60°C in a water bath, treated for 20 min with 3% nonfat dry milk at 37°C to block nonspecific antibody binding, and then incubated for 30 min with 10 *μ*g/mL monoclonal antibody anti-ssDNA (F7-26, Bender Med Systems). Finally, slides were stained for 30 min with fluorescein-conjugated anti-mouse IgM diluted 1 : 50, counterstained with 1 *μ*g/mL DAPI, and observed by a fluorescence microscope. Percentage of apoptosis was expressed as the ratio between the number of ssDNA-positive nuclei with respect to the total number of DAPI-positive nuclei.

### 2.5. Immunofluorescence Analysis

C2C12 myoblasts were seeded on round coverslides (13 mm diameter) and induced to differentiate as previously [4]. After CisPt treatment cell culture was fixed for 20 min in 4% paraformaldehyde in PBS and permeabilized for 5 min with 0.1% Triton X-100. To block nonspecific binding, slides were incubated in 10% BSA in PBS for 1 h at room temperature. Successive slides were exposed to rabbit polyclonal LC3 (1 : 200) or mouse monoclonal alpha-tubulin (1 : 1000, clone B-512) primary antibodies and then to Alexa Fluor-568 goat anti-rabbit IgG or CY3 anti-mouse secondary antibodies for 1 h (1 : 400). To counterstain nuclei we used DAPI at 5 *μ*g/mL for 10 min at room temperature. Slides were mounted with Prolong Gold antifade reagent (Molecular Probes) and maintained in a cold chamber in the dark until observation. For immunofluorescence imaging we used a confocal light microscope (CLSM 510 Meta, Zeiss) equipped with 488–568 nm laser lights.

### 2.6. Scanning Electron Microscopy (SEM)

For SEM analysis differentiated myotubes were fixed with 2.5% glutaraldehyde in 0.1 M phosphate buffer for 1 h. Then, they were air-dried and mounted on tips for the observation under a scanning electron microscope (LEO 1525 SEM, FEI Company, Eindhoven, The Netherlands) operated at 16–20 kV.

### 2.7. Transmission Electron Microscopy (TEM)

For TEM analysis, differentiated C2C12 myotubes were fixed with 2.5% glutaraldehyde in 0.1 M phosphate buffer for 15 min and then gently scraped and centrifuged at 1200 rpm to obtain pellets. They were subsequently postfixed with 1% osmium tetroxide in the same buffer, dehydrated in alcohol and propylene oxide, and then embedded in Epon 812 epoxy resin (Embed 812, Electron Microscopy Sciences, Hatfield, USA). For analysis of myofiber size and apoptosis or necrosis we used semithin sections (0.5 *μ*m thick) obtained at an Ultracut E Ultramicrotome (Leica), stained with 1% Toluidine blue or methylene blue-Azur II mixture, and observed by an immersion oil 100X objective at a light microscope (Olympus, Germany) equipped with image analysis software (Image Pro Plus, Milan, Italy). For deep characterization of autophagic vacuoles in myotubes, we collected ultrathin sections (70–90 nm thick), by a diamond blade, on formvar-coated copper grids (300 meshes), double-stained with uranyl acetate and lead citrate, and observed them under a Tecnai G2 Spirit transmission electron microscope (FEI Company, Eindhoven, The Netherlands) set at 80 kV.

### 2.8. Statistics

Quantification of myofiber size, autophagic vacuoles, or autophagolysosomes and mitochondria density were expressed as mean ± SEM as previously described [21]. LC3 puncta in multinucleated myotubes at 5 days of differentiation were quantified in each experimental treatment by using the Image J 1.44 h software (NIH, http://imagej.nih.gov/ij/). The quantification was calculated considering five randomly selected fields, each one in duplicate for each sample. Almost 50 myotubes were examined for each treatment. To compare multiple means in CisPt treated groups with DMSO-exposed control, statistical significance was determined using *t* Student's* t*-test and analysis of variance (ANOVA). A *P* value ≤ 0.05 was considered significant.

## 3. Results

### 3.1. Cisplatin Induces Progressive Myotube Disalignment, Surface Blebbings, and Atrophy

**Figure 1:**
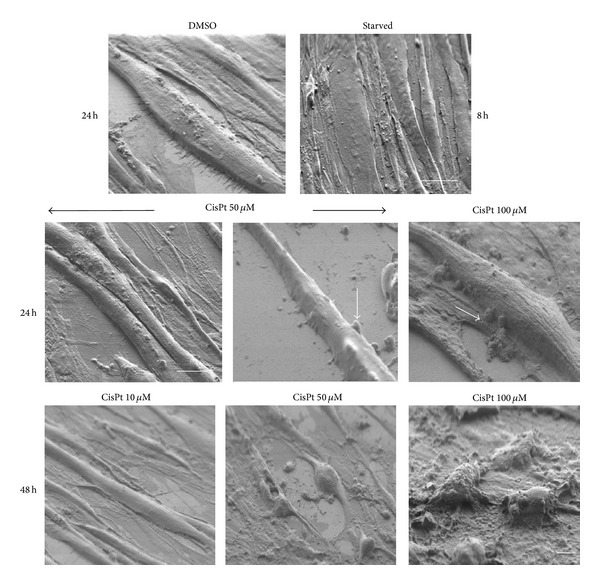
SEM microphotographs of C2C12 myotubes after DMSO, starvation, or CisPt 10, 50, and 100 *μ*M for 24 or 48 h. Note the blebbings (arrow) after CisPt 50 and 100 *μ*M, already at 24 h, and the almost complete cellular destruction at 48 h of exposure to the highest dose of the drug. DMSO, starved, and 24 h treatment Bar = 10 *μ*m and 48 h treatment Bar = 2 *μ*m.

Tridimensional skeletal muscle imaging is an important tool to best appreciate myofiber integrity that is a necessary requisite for functional efficiency. Previous studies on drug-induced muscle toxicity visualized abnormalities in the sarcolemma [22] and blebbings that are often considered early apoptotic predictors in myoblasts [23]. To best describe the sarcolemma after CisPt treatment, the presence of blebbings, the adherence of the monolayer to substrate, and the size reduction, we adopted SEM analysis that provided tridimensional pictures of myotubes and their reciprocal interactions better than contrast phase microscopy. DMSO-exposed myotubes at 5 days of differentiation were aligned in parallel arrays, almost flat and well adherent to substrate; their sarcolemma was devoid of blebbings. In contrast, starved C2C12 myotubes (adopted as positive autophagic control) showed spindle-shape myofibers where sarcolemma blebbings were evident at 8 h (Figure 1). C2C12 myotubes exposed to 10 *μ*M CisPt presented regular sarcolemma and an overall parallel orientation up to 48 h of treatment, whereas cells exposed to 50 and 100 *μ*M CisPt evidenced peculiar changes already at 24 h. In particular, myotubes lost their regular arrangement, appeared randomly oriented, and often retracted with many sarcolemma extrusions and focal reduced size (Figure 1). At increasing exposure times and doses, in particular at 100 *μ*M CisPt, overt necrosis and myotubes detachment from the substrate were detected. After 72 h of 50 and 100 *μ*M CisPt exposure, the effect on myotubes was so extreme that this model was not suitable for any further analysis (data not shown). These results were in agreement with data reported in a previous study, where apoptosis and necrosis were investigated by quantitative methods [4].

Hereafter, by employing 50 *μ*M CisPt as the ideal dose administration, subsequent experiments were addressed to study the presence of autophagy for 4 h up to 24 h in C2C12 myotubes.

### 3.2. Cisplatin Induces Autophagy in a Dose- and Time-Dependent Manner in C2C12 Myotubes

**Figure 2:**
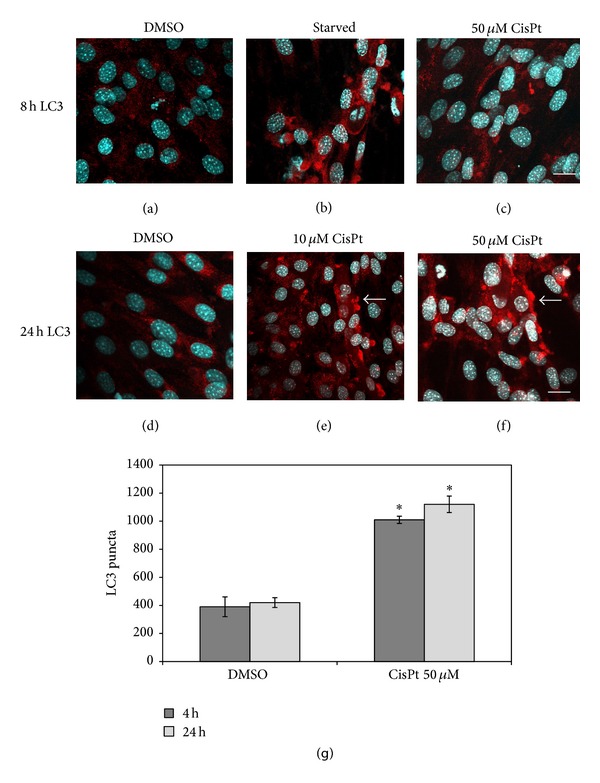
Dose-dependent LC3 distribution in C2C12 myotubes exposed to CisPt. At 10 *μ*M CisPt red spots were evident at 24 h (e), while at 50 *μ*M earlier both at 8 h (c) and at 24 h (f). For comparison starved cells at 8 h (b) and DMSO-treated cells at 8 h (a) and 24 h (d). Note the peripheral distribution of LC3-positive aggregates after 24 h of treatment with the drug. Bar = 10 *μ*m. At the bottom (g) LC3 quantification demonstrated high increase in CisPt 50 *μ*M at 4 h and 24 h versus DMSO; *P ≤ 0.05 statistically significant.

To follow autophagic vacuoles formation we analysed by immunofluorescence and confocal microscopy the presence and distribution of microtubule-associated protein 1 light chain 3B isoform I (LC3B-I). This microtubule-linked marker shifts to the different lipidated isoform II (LC3B-II) during autophagosomes and autophagolysosomes formation and has been considered a reliable autophagic marker [24]. At 4 h of 50 *μ*M CisPt exposure we detected scattered LC3-positive puncta around nucleus in myotubes similar to starved cells, used as positive autophagy control (data not shown). LC3-positive red thin spots were clearly evident even after 8 h in starved (Figure 2(b)) and in CisPt 50 *μ*M treated myotubes (Figure 2(c)). Intriguingly, upon prolonged treatments with 10 and 50 *μ*M CisPt for 24 h, C2C12 myotubes showed LC3-positive large dots scattered in the sarcoplasm (Figure 2(e)) but mainly beneath sarcolemma (Figure 2(f)). Curiously, several LC3-positive puncta were also detected in DMSO treated myotubes at 8 h up to 24 h (Figures 2(a)–2(d)), as an index of basal autophagy. This last result agreed with the peculiar behavior of autophagy observed in skeletal muscle [3, 25]: muscular cells are able to generate and maintain autophagosomes for long periods (also for days) while in other tissues the process lasts for only few hours. To best corroborate our microscopic data we quantified LC3 puncta in myotubes exposed to 50 *μ*M CisPt at 4 h and 24 h and compared data obtained with DMSO treatments (Figure 2(g)). Remarkably CisPt enhanced early and late LC3-spots distribution that quadruplicated versus respective DMSO controls. Furthermore, to validate the extent of autophagy, we added 3-methyladenine (3-MA), a known early inhibitor of autophagy machinery [20] in myotubes challenged with CisPt at 24 h, and we observed a dramatic LC3-spots reduction but increment of apoptosis and necrosis. Quantitative data relative to apoptosis or necrosis percentages in different experimental conditions were inserted in Table 1.

**Figure 3:**
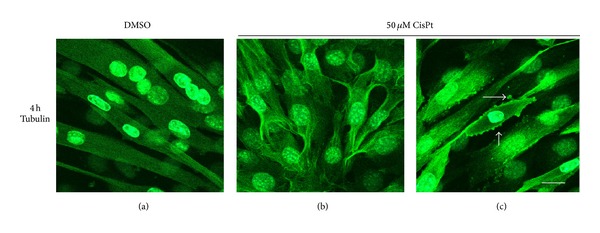
Alpha-tubulin distribution in C2C12 myotubes exposed to CisPt 50 *μ*M for 4 h. Note the anomalous organization of the microtubular network (b)-(c) versus DMSO exposed controls (a). Bar = 10 *μ*m.

It is known that a well-organized microtubular network is a fundamental prerequisite to drive the autophagosomes during onset of autophagy and then to complete their fusion with lysosomes in the final maturation step [26]. Here to best assess microtubules organization in myotubes exposed to 50 *μ*M CisPt and the impact on autophagy, we analysed alpha-tubulin distribution by immunofluorescence in treated or control muscle cells at 4 h. For this purpose we studied alpha-tubulin distribution by confocal microscopy. In DMSO-treated myotubes, alpha tubulin green network runs well-organized and parallel to sarcolemma (Figure 3(a)), while upon 50 *μ*M CisPt alpha tubulin-staining redistributed along a strict peripheral rim or in aggregates at the end of myotubes (Figures 3(b)-3(c)). This last result suggests an early damage of the machinery that would not allow the autophagosome movement from the peripheral regions of myotube to perinuclear lysosomes and might negatively influence the integrity and survival of myotubes.

**Figure 4:**
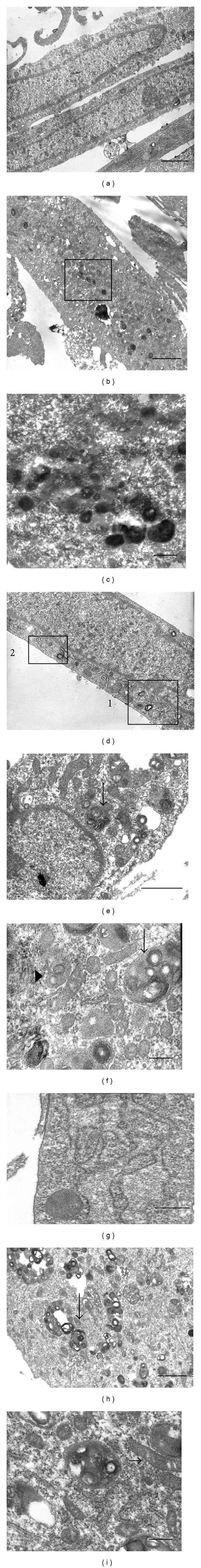
TEM micrographs of C2C12 myotubes after DMSO (a), starvation (b)-(c), CisPt 50 *μ*M at 4 h (d)–(g), and 24 h (h)-(i) exposure. Note elongated DMSO exposed controls (a), but at 4 h exposure to CisPt perinuclear autophagic vacuoles (d): at higher magnification, in square 1, in the same myotube such structures resembled autophagosomes (arrowhead) and autophagolysosomes (arrow) (e)-(f), and, in square 2, enlarged endoplasmic reticulum (g). Under 50 *μ*M CisPt for 24 h clusters of autophagolysosomes (arrow) near the sarcolemma (h-i). In starved myotubes, irregular electron dense deposits (b) that at higher magnification (squared area) resembled secondary lysosomes and osmiophilic membranous debris (c). (a), (b), (e), and (h) Bar = 1 *μ*m; (c), (f), (g), and (i) Bar = 0.5 *μ*m.

To best characterize autophagic vacuoles and their different features along the progression of autophagy, we performed TEM analysis, recognized as the gold method to detect and monitor autophagy in mammalian cells [27, 28]. C2C12 myotubes treated with DMSO appeared elongated with multiple nuclei with characteristic indentations and regular organelles (Figure 4(a)), while after 4 h at 50 *μ*M CisPt exposure, autophagic vacuoles, myelinic whorls, autophagolysosomes, and dilatation of endoplasmic reticulum were observed around nuclei (Figures 4(d)–4(g)). Moreover, starting from 8 h up to 24 h of CisPt treatment, autophagolysosomes appeared largely filled with heterogeneous materials and often associated with scattered round and dense mitochondria with amorphous cristae; finally, at 24 h, several clusters of autophagic vacuoles, osmiophilic myelinic figures that migrated at the end of the fibers or beneath the sarcolemma, and apoptotic nuclei with condensed chromatin were detected (Figures 4(h)-4(i)). Interestingly, starved myotubes showed irregular amorphous dense bodies and membranous debris located inside of sarcoplasm (Figures 4(b)-4(c)).

### 3.3. Taurine Supplementation Rescues Cisplatin-Induced Muscle Atrophy at 24 h

**Figure 5:**
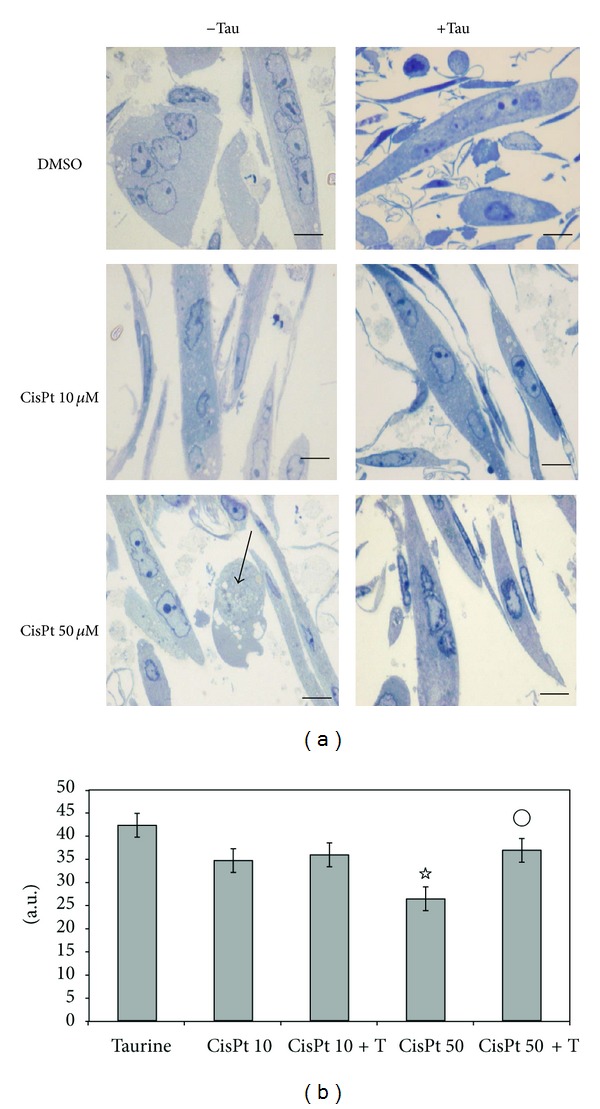
(a) Toluidine blue-stained C2C12 myotubes exposed for 24 h to DMSO, 10 *μ*M, or 50 *μ*M CisPt alone or combined with Tau. Tau preserved morphology like in DMSO and 10 *μ*M CisPt treatments, but atrophy induced by 50 *μ*M CisPt was restored by Tau. Arrow indicated necrosis. Bar = 20 *μ*m. (b) Analysis of C2C12 myotubes' size exposed to CisPt in the presence or not of Tau. _ _^☆^ *P* ≤ 0.05 compared with DMSO; °*P* ≤ 0.05 compared with CisPt 50 *μ*M (*n* = 3).

In order to investigate the effect of Tau on myotube atrophy we added 25 mM Tau or vehicle to the media containing or not 10 or 50 *μ*M CisPt doses; we analyzed myotube cultures by light microscopy or TEM after 24 h from the beginning of treatment. Tau concentration was in a range 20 mM–60 mM effective against CisPt-induced damage in other cell types or in muscle disorders [16, 29]. Remarkably Tau pretreatment did not affect myotubes, which typically appeared in regular arrays with multiple indented nuclei (Figure 5(a)). However, after 10 *μ*M CisPt exposure only few cells became occasionally thinner and their overall shape did not change significantly after Tau supply. In contrast, after 50 *μ*M CisPt for 24 h myotubes dramatically changed size and apoptosis or necrosis occurred. Interestingly, when Tau was added to 50 *μ*M CisPt, the majority of atrophic myotubes became almost similar to controls (Figure 5(a)). Computations of myotube size demonstrated that with respect to Tau alone or DMSO controls, there was a low reduction (about 10%) in 10 *μ*M CisPt that was maintained also under Tau pretreatment; in contrast 50 *μ*M CisPt significatively affected cell size (reduced at 40%) and Tau supplementation recovered myotube size at almost control value. All quantitative data as mean ± SE were plotted in Figure 5(b).

**Figure 6:**
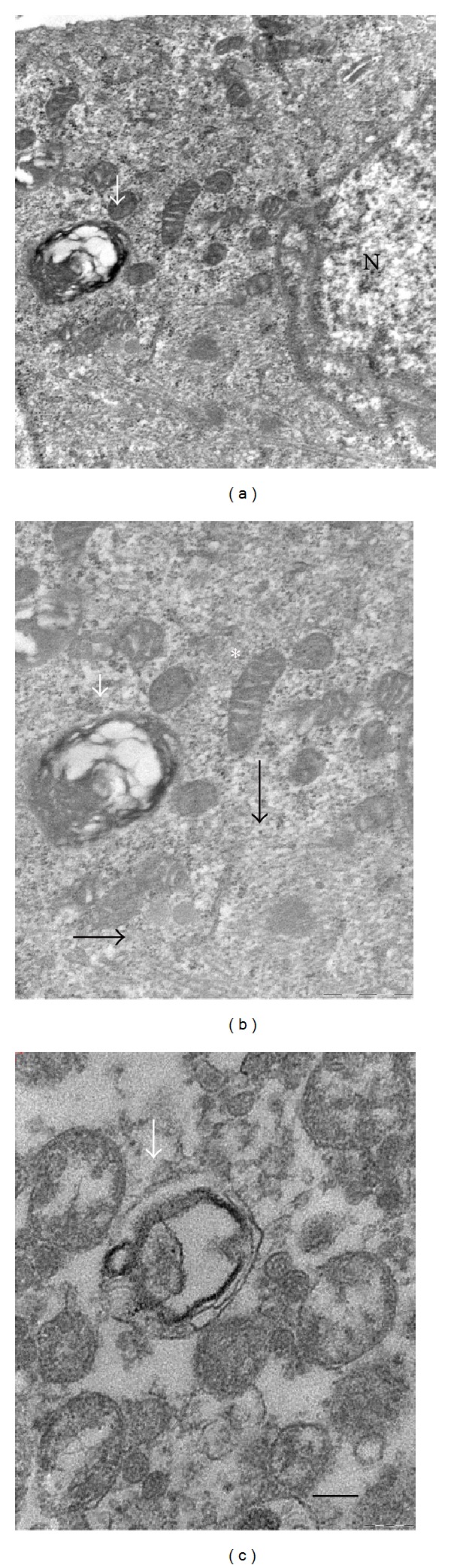
Ultrastructure of C2C12 myotubes challenged with 50 *μ*M CisPt for 24 h in the presence of Tau. (a) Note mitochondria and autophagolysosome around nucleus (N); (b) at higher magnification, autophagosomes, well-organized microtubules (white arrows), and mitochondria with regular cristae (asterisk); (c) destroyed mitochondrion inside an autophagic vacuole (dark arrow) in the culture medium together with vesicles and cellular debris. Bar = 200 nm.

Furthermore, to best address the influence of Tau on autophagy, we performed TEM analysis in C2C12 myotubes treated as shown above for 24 h. Although in 10 *μ*M CisPt exposed cells Tau preconditioning did not induce overt autophagic or apoptotic figures (data not shown), cells challenged with 50 *μ*M CisPt and Tau presented less autophagolysosomes in the sarcoplasm but surprisingly similar figures and myelinic debris were detected in the medium. Even if with a mechanism yet unknown, this last finding may indicate an intense clearing of the sarcoplasm by Tau. Moreover, blebbings associated to CisPt apoptotic damage disappeared and a regular microtubular network and round and elongated mitochondria with well-preserved cristae were observed in the sarcoplasm (Figures 6(a)–6(c)).

## 4. Discussion

Cisplatin (CisPt) is a widely used cytostatic drug that stimulated both apoptosis and autophagy in different normal and cancer cell types [30]. Among its multiple subcellular targets, besides nucleus, there are mitochondria, cytoskeleton, endoplasmic reticulum, and lysosomes; all involved in a complex interplay in autophagy or apoptosis processes [31, 32]. In skeletal muscles, autophagy is starting to emerge only in these last years: it appears to be necessary for protein and organelles renovation and represents a novel therapeutic target in Duchenne muscular dystrophy [33]. Indeed abnormal autophagy has been associated with disorders characterized by accumulation of abnormal mitochondria or inclusions, excessive physical activity, and cancer cachexia [34–36].

In the current microscopic study we visualized the onset and progression of autophagy in C2C12 myotubes treated with 50 *μ*M CisPt from 4 h to 48 h and its relationship with atrophy generated by this drug. Interestingly we reported that 25 mM Tau preconditioning prevented CisPt-induced atrophy and restored proper autophagic vesicles, mitochondria cristae, and regular microtubular apparatus.

Our novel morphological data in skeletal myotubes are interesting for different reasons: first of all, they confirmed the presence of a basal persistent autophagy in DMSO control myotubes at 24 h as an adaptive and cytoprotective reaction to intense catabolism in the muscle [3, 25]; second, upon 50 *μ*M CisPt early new autophagy was further superimposed to basal autophagy but was impaired at 24 h when excessive autophagolysosomes accumulated and apoptosis occurred; finally, different from CisP-treated, starved myotubes showed up to 8 h peculiar autophagic vacuoles, very similar to secondary lysosomes filled with lipidic debris. Afterwards prolonged starvation induced massive destruction of myotube constituents, so that excessive autophagy resulted in detrimental effect [37, 38]. It is known that a hypercatabolic status induced by nutrient deprivation or by CisPt may produce different inclusion bodies as signs of inhibition of two main catabolic systems, ubiquitin-proteasome and autophagy-lysosome pathways [39]. Because of their specific role, both cardiac and skeletal muscles must sustain an intense protein renovation and synthesis to fulfill energy requirements and regulate inner homeostasis [40]. So as a direct consequence of high oxidative metabolism in skeletal muscle, an adequate autophagy, the recycling, and the elimination of cell debris are imperative. We demonstrated here that CisPt potentiated the basal autophagic activity in myotubes by increasing LC3-positive dots from 4 h up to 24 h. Up to 8 h, nuclei were regular and devoid of apoptotic signs, in line with Xiao et al. [41] and Salucci et al. [42] who reported resistance to apoptosis challenge in this differentiated in vitro system. Therefore, in the first 24 h of treatment C2C12 myotubes appeared to react against CisPt insult by activating the autophagy-lysosome system that preserves cell integrity and function. Later, clear traces of apoptosis, such as chromatin condensation, became evident, suggesting an impairment of the autophagic pathway. Intriguingly, we demonstrated here that when autophagic pathway is stopped by a specific inhibitor (3-MA), consistent apoptosis and necrosis occurred even at 24 h, when generally these phenomena are reduced.

A regular microtubular apparatus is mandatory for autophagic process, because early phagophores have to move along cytoskeletal structures to accomplish their fusion with lysosomes and form autophagosomes and mature autophagolysosomes [20]. If these final autophagic figures exceed the onset of new autophagosomes, myotubes are progressively engulfed by heterogeneous waste that was not recycled nor digested. Peculiarly, we evidenced that 50 *μ*M CisPt affected tubulin organization already at 4 h of treatment. The microtubular network disorganization and the peculiar location of alpha-tubulin near the sarcolemma might be responsible for the accumulation of mature autophagic vacuoles observed at 24 h. Although upon 50 *μ*M CisPt challenge at 4 h an intense detoxifying input was evident driven by ER activation and the onset of perinuclear autophagosomes and at 24 h the system became unable to remove debris, damaged mitochondria, and cytoskeleton. So abnormal autophagolysosomes near peripheral sarcolemma and blebbings represent signs of failed exocytosis, dysfunctional autophagy, apoptosis, or a cross-reaction between myotubes in an attempt to resolve such a metabolic impairment. The peculiar ultrastructure of the majority of vesicles filled with amorphous and lamellar material resembled polyubiquitinated deposits described in muscles of autophagy-deficient Pompe mice [43].

In addition, we firstly demonstrated by morphological and morphometric methods that Tau acts as an effective regulator of autophagic progression and improves CisPt-induced atrophy in murine C2C12 myotubes. While Tau was not so effective at 10 *μ*M CisPt treatment, probably due to the absence of significant atrophy and a well-preserved myofibrillar apparatus at this very low dose [4], when added to 50 *μ*M CisPt, myotubes were cleared by dysfunctional mature autophagic vacuoles and membranous debris. In particular 25 mM Tau, besides maintaining myotube size, was also able to protect mitochondria cristae and microtubular apparatus by the toxic action of CisPt. Considering that oxidative metabolism greatly regulates muscles' activity to provide adequate energy necessary to their functions, mitochondria integrity in myotubes is a fundamental requisite. It has been demonstrated that mitochondria-targeted drugs represent a potent response against CisPt renal damage [44].

Tau has been reported to have a wide developmental potential and cytoprotective functions in skeletal muscle where it is delivered by Tau transporter (Tau T) [45]. Indeed in TauT-knockout rodents, several cardiac and skeletal muscle alterations occurred [46, 47] while Tau T expression was effective against dexamethasone-induced atrophy in vitro [48]. Tau was able to prevent oxidative damage, ER stress, and apoptosis in cardiomyocytes [49, 50] and we recently reported that Tau rescued CisPt apoptosis in renal cells by inducing autophagy and limiting ER stress [16]. Many recent studies in different cell types indicated that CisPt activated apoptosis by affecting mitochondria and cytoskeleton that were associated to intrinsic apoptotic pathway [51]. On the other hand, Tau has been reported to be effective against apoptosis in neurons by blocking caspase 3-pathway and restoring microtubule associated protein 2 (MAP-2) [52]. Nevertheless, Tau is able to potentiate CisPt anticancer activity in human cervical cancer cells, where it accelerated apoptotic events [53]. So the complex action of Tau, pros or contra CisPt, must be strictly evaluated in different cell types and experimental conditions.

## 5. Conclusion

In conclusion, we proved by a multidisciplinary morphological approach that in vitro murine myotubes exposed to increasing CisPt doses showed excessive autophagy, relatively late apoptosis, and atrophy that occurred at high doses and long times of exposure (24 h). Autophagy occurred very early in 50 *μ*M CisPt challenged myotubes (at 4 h–8 h) before overt atrophy but curiously persisted even at 24 h, when several autophagolysosomes appeared in the sarcoplasm together with damaged mitochondria and sarcoplasmic blebbings. The autophagic machinery seemed necessary as an adaptive response to persistent catabolism and fulfills the requirement of organelle renovation. Indeed specific autophagy impairment by 3-MA accelerated unavoidable apoptosis or necrosis. Nevertheless, Tau pretreatment before 50 *μ*M CisPt preserved the size of the majority of myotubes at 24 h. We can only speculate that Tau improves CisPt-induced atrophy by reducing autophagosomes overload and restoring regular microtubular apparatus and mitochondria.

Tau is an essential regulator of intracellular osmolarity and influences calcium flux and membrane exchanges. However, even if the mechanism of Tau regulation of atrophy and autophagy in our muscle model is currently unknown, several morphological evidences indicate that this free beta-amino acid acts on cellular CisPt targets, essential to maintain the autophagic flux. This is in line with Bursch et al. [54] who considered autophagy in skeletal muscles important to avoid atrophy but only if properly conducted.

Finally, even if Tau pretreatment seems promising to rescue from atrophy and dysfunctional autophagy in C2C12 model, further studies on this topic are mandatory not only in vitro but also specifically in vivo, where different components, such as innervation, hormones, blood circulation, or exercise, modulate the physiological response of skeletal muscles, greatly modifying the observed Tau protective effects.

## Figures and Tables

**Table 1:** Apoptosis and necrosis percentages in C2C12 myotubes exposed for 24 h to increasing concentration of CisPt in the presence or not of 3-methyladenine (3-MA).

Treatment	Apoptosis (mean ± SE)	Necrosis (mean ± SE)
DMSO	1.7 ± 0.2	n.d.
CisPt 10 *μ*M	1.9 ± 0.5	n.d.
CisPt 50 *μ*M	3.1 ± 0.9°	n.d.
DMSO + 3-MA	1.9 ± 0.6	n.d.
CisPt 10 *μ*M + 3-MA	31.4 ± 1.6^∗°^	2.4 ± 0.8^∗°^
CisPt 50 *μ*M + 3-MA	37.5 ± 4.3^∗°^	5.1 ± 0.2^∗°^

n.d.: not detected; *P ≤ 0.05 statistically significant with respect to CisPt 50 alone treatment; °*P* ≤ 0.05 statistically significant with respect to DMSO.
